# Effect of Various Body and Head Positions on Intraocular Pressure in Cataract Patients With Limited Mobility

**DOI:** 10.1155/joph/2019418

**Published:** 2025-09-02

**Authors:** Yanxia Tong, Jing Yuan, Tingting Peng, Huafang Guo, Biyue Tu, Haifeng Jiang, Yong Wang

**Affiliations:** ^1^Department of Ophthalmology, Renmin Hospital of Wuhan University, Wuhan University, Wuhan, Hubei, China; ^2^Aier Eye Hospital of Wuhan University (Wuhan Aier Eye Hospital), Wuhan University, Wuhan, Hubei, China

**Keywords:** immobile, intraocular pressure, posture

## Abstract

**Purpose:** To assess the impact of various body and head positions on intraocular pressure (IOP) in cataract patients aged over 40 years with limited mobility.

**Methods:** This cross-sectional study was conducted between August and December 2023 at Aier Eye Hospital of Wuhan University. The IOP was measured using a handheld tonometer (iCare IC200 rebound tonometer) in various head positions (forward, tilted left, and tilted right) and body positions: supine, semirecumbent, sitting, and prone.

**Results:** In the supine position, the IOP measurements for the head positioned forward, right, and left were (13.80 ± 3.62) mmHg, (14.25 ± 3.66) mmHg, and (13.78 ± 3.40) mmHg, respectively. In the semirecumbent position, the corresponding IOPs were (12.08 ± 3.34) mmHg, (12.12 ± 3.22) mmHg, and (12.04 ± 3.38) mmHg. In the sitting position, the IOPs were recorded as (11.73 ± 3.29) mmHg, (11.73 ± 3.22) mmHg, and (11.59 ± 3.17) mmHg. Lastly, in the prone position, the IOPs were (14.19 ± 3.73) mmHg, (14.42 ± 3.93) mmHg, and (14.74 ± 3.81) mmHg, respectively. In each position group, there was no statistically significant difference in IOP among the three head positions. Regardless of the head position, the IOP is lowest in the sitting position, followed by semirecumbent and supine positions, with the prone position having the highest IOP. The analyses revealed that central corneal thickness (CCT) was correlated with an IOP value (*p* < 0.05) when patients were in different positions.

**Conclusion:** IOP is influenced by body position. As the body transitions from upright to horizontal, IOP tends to increase. The position of the head, however, has no effect on IOP.

## 1. Introduction

Intraocular pressure (IOP) is an important physiological parameter for evaluating eye health and disease. It represents a crucial vital sign of the eye. Along with visual acuity, pupillary examination, and visual field test, measuring IOP in a consistent and reliable manner is fundamental to the diagnosis and treatment of glaucoma and related diseases [1]. Both ocular hypertension and ocular hypotension are associated with various ocular pathologies. Some cataract patients experience lens expansion in the later stages of cataract, leading to increased IOP, which can be timely detected through IOP measurement to prevent irreversible damage to the optic nerve from glaucoma [2]. Consistency in IOP measurements is critical to the accuracy of diagnosis and therapy. However, IOP measurements can be affected by different patient positions or different types of IOP measuring devices [3]. Many daily activities, such as yoga, Valsalva maneuver and varying sleep positions, can lead to transient increases in IOP [4]. However, some of the temporary fluctuations in IOP during daily activities are difficult to capture, such as those during heavy lifting or running. As a result of varying lifestyle habits, different patients may spend extended periods in positions that favor an increase in IOP. This could potentially influence the prognosis of glaucoma and related diseases [5]. Therefore, it is urgent to evaluate the IOP in different postures for clinical diagnosis and treatment.

In clinical practice, rebound tonometers offer a more user-friendly IOP measurement experience for patients in special positions, such as those in a coma, paralyzed, or other difficult-to-measure positions, or children who cannot cooperate with the examination [6–9]. This study aims to analyze the effects of different body positions and head positions on IOP. In this study, the iCare IC200 was utilized to measure IOP in various body positions and head positions, to assess its consistency and to investigate the factors that cause IOP changes with body positions.

## 2. Methods

### 2.1. Study Design

This cross-sectional study was conducted between August and December of 2023. The IOP was measured using a handheld tonometer (iCare IC200 rebound tonometer) with the head positioned forward, left, and right in the supine, semirecumbent, sitting, and prone positions. All participants provided informed consent before participating in the study. We adhered to all ethical standards, ensuring the confidentiality and privacy of all patient data. All participants' information was anonymized and securely stored. Data collection and handling were performed in accordance with the principles of the Declaration of Helsinki and relevant data protection regulations. We took all necessary measures to protect patient privacy and ensure the secure handling of sensitive information throughout the study.

### 2.2. Subjects

The inclusion criteria were as follows [10]: (1) patients with cataract are immobile or bedridden and (2) those sitting IOP within the normal range (≤ 21 mmHg, where 1 mmHg = 0.133 kPa) and undergoing a detailed ophthalmologic examination.

The exclusion criteria were as follows [11]: (1) patients with any type of glaucoma (including treated glaucoma patients); (2) those with ocular conditions that prevent IOP measurement, such as corneal inflammation, corneal edema, and corneal scars; (3) those with history of corneal refractive surgery; (4) those with conditions other than glaucoma that could affect IOP assessment, such as larger intraocular or orbital masses and iris neovascularization; (5) those with history of ocular surgery within the past 3 months; and (6) those with the inability to provide informed consent for various reasons.

### 2.3. IOP Measurement Method

**Figure 1:**
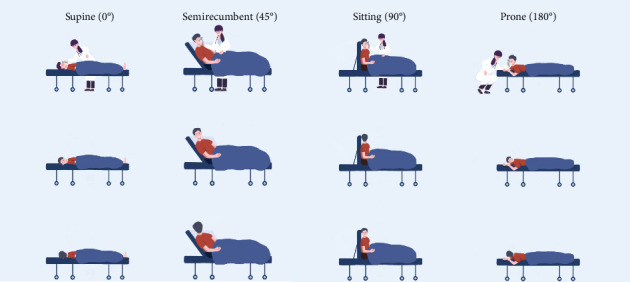
Different positions for measuring intraocular pressure.

A rebound tonometer (iCare IC200 Rebound Tonometer TA031, iCare FinlandOy, Finland) was used as the measuring device, ensuring accuracy and stability. IOP was recorded from one eye for each participant. Enrolled participants were randomized using an interactive web response system (https://randomizer.org) in a 1:1 ratio to select the study eye. All examinations were performed by two different examiners in a dedicated examination room between 1:00 p.m. and 3:00 p.m. All subjects had their pupils in a natural state. IOP was measured in supine (0°), semirecumbent (45°), seated (90°), and prone (180°) positions. Each position consisted of three head positions: head-forward, head-right, and head-left (Figure 1). First, each participant was asked to sit on a backrest chair in a quiet room under dim light conditions. Five minutes later, IOP was measured in the sitting position with three head positions. For the random sequence of the recumbent positions, each participant was asked to pick up one card, where supine, semirecumbent, or prone positions was written on the back, one after another until all the three cards were open. According to the order of the cards chosen, each participant was informed of the sequence of the recumbent postures. Each position was maintained for 5 min before measurement, and the patients had taken a 5-min break between measuring IOP in different postures. For each IOP measurement with the iCare tonometer, 6 consecutive readings were taken, discarding the highest and lowest values to calculate the average of the remaining four measurements. The average IOP was displayed in millimeters of mercury (mmHg) to one decimal place. The iCare tonometer indicated low variation with a green display, and high variation with a yellow display. If there was unacceptably high variation, a repeat symbol was shown. Readings with CV > 15% across 3 consecutive measurements at the same posture were discarded and remeasured. For this study, only values displayed in green were used. The results of each measurement, averaging six or more measurements taken in each eye, were recorded. Subjects missing valid data in any posture were excluded from the analysis (0 cases were excluded in this study). Axial length (AL), central corneal thickness (CCT), anterior chamber depth (ACD), lens thickness (LT), and mean keratometry (Km) were measured by Master 700 (Carl ZeissMeditec AG, Jena, Germany).

### 2.4. Statistical Methods

Sample size was calculated with G∗Power software (Version 3.0.10, Universita¨t Kiel Dusseldorf, Germany), and we used *α* = 0.05 and *β* = 0.2. A sample size calculation determined that 50 eyes would be required to detect an IOP difference during postural change with a power of 80%. The final sample size (*n* = 150) exceeded the calculated requirement (*n* = 50), ensuring higher statistical power.

Although we primarily analyzed data per-eye, the potential dependency was rigorously addressed with intraclass correlation coefficient (ICC) quantification. The intereye ICC for postural IOP changes was 0.08 (95% CI: −0.12∼0.28, *p*=0.41), below the significant threshold (ICC > 0.3) for ophthalmologic studies.

The SPSS 25.0 software package was used for data processing. The measurement data such as IOP values were tested for normality. The continuous variables conforming to the normal distribution were described by the mean and standard deviation ($\overset{¯}{x}\pm s$). Analysis of variance (ANOVA) was performed after verifying the assumptions of normality. Repeated-measures ANOVA with Bonferroni post hoc tests are applied. The mean IOP values of different positions were compared, and the ICC was utilized to assess the consistency of the mean IOP values across positions. The nonparametric Spearman's correlation test and Wilcoxon signed-rank test were both employed to statistically evaluate relationships between the participants' age, gender, and the different body positions. *p* values < 0.05 were considered statistically significant.

## 3. Results

### 3.1. Demographic Data

One hundred and fifty participants with 150 eyes were included in this study. The mean patient age was 69.33 ± 10.23 years (range: 40–87 years). There were 60 (40%) male and 90 (60%) female participants.

### 3.2. Relationship Between Body Position and IOP

**Figure 2:**
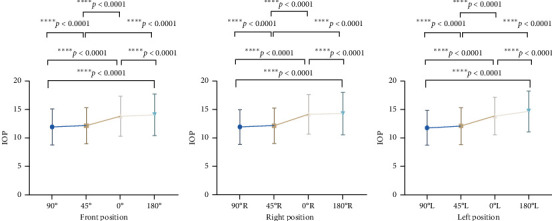
Intraocular pressure values for different positions and head positions.

The mean IOP values measured in different head positions of the patients were highest in the prone position, followed by supine, semirecumbent, and lowest in the sitting position (Figure 2). In the forward head position, the IOP was (14.19 ± 3.73) mmHg in the prone position, (13.80 ± 3.62) mmHg in the supine position, (12.08 ± 3.34) mmHg in the semirecumbent position, and (11.73 ± 3.29) mmHg in the seated position. The difference in IOP between the four positions was statistically significant (*p* < 0.0001). There was good agreement in IOP across the four groups of positions, with ICC = 0.801 when the head was in the forward position (*p* < 0.0001).

### 3.3. Relationship Between Head Position and IOP

In the supine position (Table 1), IOPs for head forward, right, and left were (13.80 ± 3.62) mmHg, (14.25 ± 3.66) mmHg, and (13.78 ± 3.40) mmHg, respectively, showing no significant difference (*p* > 0.05). In the semirecumbent position, IOPs were (12.08 ± 3.34) mmHg, (12.12 ± 3.22) mmHg, and (12.04 ± 3.38) mmHg, with no significant difference (*p* > 0.05). In the sitting position, IOPs were (11.73 ± 3.29) mmHg, (11.73 ± 3.22) mmHg, and (11.59 ± 3.17) mmHg, respectively, again with no significant difference (*p* > 0.05). In the prone position, IOPs were (14.19 ± 3.73) mmHg, (14.42 ± 3.93) mmHg, and (14.74 ± 3.81) mmHg, also showing no significant difference (*p* > 0.05).

### 3.4. Relationship Between Biometric Parameters and IOP

Table 2 presents the results of the nonparametric Spearman's correlation test between each IOP and variables, AL, CCT, ACD, LT, and Km. The analyses demonstrated that CCT was correlated with the IOP value (*p* < 0.05) when patients were in the supine, semirecumbent sitting, and prone positions. Other ocular biological parameters were not significantly correlated with IOP values. All IOP values in the current study measured by iCare IC200 were affected by the CCT.

### 3.5. Relationship Between Gender, Age, and IOP

The relationship between the participants' gender and IOP in different body positions was examined using the Wilcoxon test (Table 3), and the participants' age distribution and IOP in different body positions were examined using Spearman's correlation test (Table 4).

No relationship was observed between age and IOP measured in supine, semirecumbent, sitting, and prone positions (*p*=0.122, *p*=0.144, *p*=0.348, and *p*=0.144).

Relationships between gender and IOP measured in supine, semirecumbent, sitting, and prone positions were statistically significant according to the results of the Wilcoxon test (*p* = 0.006, *p* = 0.006, *p* = 0.028, and *p* = 0.001).

## 4. Discussion

IOP is an important physiological parameter for assessing ocular health and disease. Measurements taken of patients in different positions and with different types of tonometers can affect IOP readings [12]. Consistency in IOP measurement is critical to diagnostic and therapeutic accuracy. The rebound tonometer (iCare IC200 Rebound Tonometer TA031, iCare FinlandOy, Finland) is a recently developed handheld device for IOP measurement. Introduced in 2020, it weighs approximately 260 g, has a probe size of 1.8 mm, and is capable of measuring IOP between 7 and 50 mmHg with good reliability [13]. The iCare IC200 tonometer showed good agreement with the Goldmann applanation tonometer (GAT) [7–9]. In an Indian population, Badakere et al. [12] found that the iCare IC200 overestimated the GAT IOPs by 1.3 mmHg. In a Japanese population, Nakakura et al. [14] found that the iCare PRO and the iCare IC200 underestimated IOPs by an average of 1 and 3 mmHg, respectively.

Changes in body position can cause many kinds of hydrodynamic changes, and these changes maybe the main reason for the increase in IOP [12]. Previous studies have shown that IOP measured in the supine position is higher than that measured in the sitting position, regardless of the type of tonometer used [15]. This is true in the general population, ocular hypertension population, or glaucoma patients [16]. These findings are similar to our results. Unlike previous studies, however, this study measured IOP in sitting, supine, semirecumbent, and prone positions, as well as in head forward, right, and left positions, providing a more comprehensive evaluation of the consistency of IOP measurements in different positions and head orientations. We found that the average IOP measured in different head orientations was highest in the prone position, followed by the supine, semirecumbent, and sitting positions (*p* < 0.0001). In the same head position, as the patient's position changed from sitting to horizontal supine, or from sitting to semirecumbent and supine, the IOP value gradually increased with this positional change. The IOP values measured in different postures were in good agreement, although changes in position may cause IOP changes. At the same time, this study also found that there was no significant difference in IOP between different head orientations in the same body position.

We found that head orientation (forward, right, and left) does not significantly affect IOP. Whole-body posture change induces jugular venous congestion 8–10 mmHg, whereas isolated head motion causes < 1 mmHg central venous pressure fluctuation due to jugular valve buffering [17].

Previous reports have indicated that IOP values of sitting and supine were affected by the CCT [18]. In the current study, IOP was measured using iCare IC200 in 150 eyes of patients with cataract. The supine and semirecumbent IOP values were affected by the CCT (*r* > 0.20, *p* < 0.05), which agrees with the findings of previous studies using IC200.

There are two main reasons for the change in IOP caused by body position changes. Firstly, ocular artery pressure and intraocular vascular volume change, affecting IOP [14–16]. Shifting from sitting to lying increases venous return, raising cardiac output and ocular arterial pressure, which enlarges the choroidal vascular bed [16, 19]. Gravity also increases head blood volume, elevating ocular perfusion pressure, venous pressure, and intraorbital pressure, thereby increasing aqueous humor drainage resistance and IOP [20, 21]. The dominant eye faces greater gravity in lateral positions, further raising IOP. Nelson et al.'s mathematical model confirmed that gravity-driven hydrostatic pressure changes acutely influence IOP, emphasizing gravity's role in IOP fluctuations [12]. Secondly, changes in superior scleral venous pressure affect IOP [21, 22]. When superior scleral venous pressure rises above the IOP level, blood flows back into Schlemm's canal, resulting in obstruction of aqueous humor outflow [23]. In addition to indirect effects, Plotnikov et al. also showed that changes in blood pressure can directly affect changes in IOP, with an increase in IOP of 0.28 mmHg for every 10 mmHg increase in systolic blood pressure [19].

In clinical practice, the influence of patient's posture on IOP should be considered. Measuring IOP in both the sitting and supine positions of patients can evaluate IOP control more comprehensively, which will be beneficial to the diagnosis and treatment of glaucoma. Health education for glaucoma patients should guide patients to avoid prone position and lateral position, which is conducive to controlling IOP.

A limitation of this study is that it did not simultaneously collect data on IOP value changes corresponding to increased duration in maintained positions. While positional changes in IOP were investigated, this aspect requires further exploration. In future studies, the time factor will be incorporated in order to observe the relationship between extended duration and IOP values. The present study only measured the changes in IOP values in different body positions in the healthy population. We excluded patients with glaucoma. It aims to avoid the potential impact of glaucoma treatments, such as medication or surgery, on IOP. Patients with glaucoma are typically more sensitive to changes in IOP, and their mechanisms for regulating ocular pressure may differ from those of healthy individuals. Consequently, the results of this study may not be directly generalizable to the glaucoma patient population. For these patients, the effects of posture changes on IOP maybe more pronounced or complex, necessitating further specialized research to investigate. We also excluded patients who had recently undergone ophthalmic surgery, which may limit the generalizability of the study results to patients with a history of such surgeries. Although this study provides valuable insights into the impact of different postures on IOP, the biases inpatient selection and exclusion criteria may restrict the generalizability of the findings to a broader population. Particularly, the results of this study may not have direct applicability to glaucoma patients and those who have recently undergone ophthalmic surgery. In the future, it would be beneficial to recruit more patients with high IOP to better explore its effect on ophthalmic diseases such as glaucoma. To further explore the impact of posture changes on IOP, we suggest that future studies could combine longitudinal and cross-sectional approaches to gain a more comprehensive understanding of the effects of posture changes on IOP. Longitudinal studies are helpful in revealing causal relationships and long-term effects, while cross-sectional studies are suitable for quickly assessing IOP differences among various populations. Future research should consider including a more diverse population, especially high-risk groups and specific occupational groups, to obtain broader and more in-depth research results. Additionally, other internal and external factors are known to influence IOP. For example, it is well established that during esophagogastroduodenoscopy, transient changes in body position and systemic pressure can significantly affect IOP [24]. Furthermore, body mass index has been shown to contribute to IOP variation in different body positions. These aspects further support the broader clinical relevance of investigating IOP dynamics in various settings.

The IOP values, as found in this study, were correlated with body position and increased as the tilt of the body position increased from upright to horizontal, with the lowest IOP in the seated position and the highest IOP in the prone position. IOP values were not related to the head position.

## Figures and Tables

**Table 1:** Intraocular pressure values in different positions and angles.

	Front (mmHg) ± SD	Right (mmHg) ± SD	Left (mmHg) ± SD	*p*
0° supine	13.80 ± 3.62	14.25 ± 3.66	13.78 ± 3.40	> 0.05
45° semirecumbent	12.08 ± 3.34	12.12 ± 3.22	12.04 ± 3.38	> 0.05
90° sitting	11.73 ± 3.29	11.73 ± 3.22	11.59 ± 3.17	> 0.05
180° prone	14.19 ± 3.73	14.42 ± 3.93	14.74 ± 3.81	> 0.05
*p*	**0.0001**	**0.0001**	**0.0001**	

*Note:* Bold values indicate statistical significance at *p* < 0.0001.

**Table 2:** Effects of axial length, anterior chamber depth, lens thickness, white to white, corneal curvature, and central corneal thickness on IOP measurement.

Parameter	0° supine		45° semirecumbent		90° sitting		180° prone	
	Coefficient	*p* value	Coefficient	*p* value	Coefficient	*p* value	Coefficient	*p* value
Axial length	0.018	0.844	0.165	0.085	0.160	0.084	0.140	0.131
Anterior chamber depth	0.189	0.059	0.151	0.136	0.163	0.105	0.146	0.147
Lens thickness	−0.115	0.196	−0.162	0.069	−0.088	0.327	−0.096	0.285
Corneal curvature	−0.131	0.156	−0.132	0.154	−0.090	0.335	−0.131	0.157
Central corneal thickness	**0.183**	**0.045**	**0.177**	**0.046**	**0.215**	**0.018**	**0.205**	**0.032**

*Note:* Bold values indicate statistical significance at *p* < 0.05.

**Table 3:** Relationship between gender and intraocular pressures measured in supine, semirecumbent, sitting, and prone positions.

	0° supine	45° semirecumbent	90° sitting	180° prone
*Z*	−2.743	−2.725	−2.191	−3.225
*p*	0.006	0.006	0.028	0.001

**Table 4:** Relationship between age and intraocular pressures measured in supine, semirecumbent, sitting, and prone positions (Spearman correlation).

	0° supine	45° semirecumbent	90° sitting	180° prone
Coefficient	−0.138	−0.13	−0.084	−0.13
*p*	0.122	0.144	0.348	0.144

## Data Availability

The data of this study are available from the corresponding author (Yong Wang) upon reasonable request. However, due to containing sensitive personal information of patients, access to the data requires approval from the institutional ethics committee of this institution and the signing of a data confidentiality agreement.
